# Seasonal and photoperiodic effects on lipid droplet size and lipid peroxidation in the brown adipose tissue of bank voles (*Myodes glareolus*)

**DOI:** 10.1007/s13364-012-0083-z

**Published:** 2012-05-18

**Authors:** Elżbieta Bonda-Ostaszewska, Tadeusz Włostowski, Alicja Krasowska, Paweł Kozłowski

**Affiliations:** Institute of Biology, University of Bialystok, Świerkowa 20B, 15-950 Białystok, Poland

**Keywords:** Bank vole, Brown adipose tissue, Lipid droplets, Lipid peroxidation, Photoperiod

## Abstract

Seasonal changes in lipid droplet size and lipid peroxidation in the brown adipose tissue (BAT) of wild bank voles were examined. In addition, a role of photoperiod in these changes was studied; bank voles were held from the birth under long photoperiod (LP) for 12 weeks, and then half of them was transferred to short photoperiod (SP) for 6 weeks and another one remained under LP. In the wild bank voles the absolute BAT weight was seasonally constant, while the significant differences in the lipid droplet size were observed. The smallest lipid droplets (mean, 11 μm^2^) were seen in winter; they increased by 30 % in spring and reached the highest size (24 μm^2^) in summer. Lipid peroxidation in the BAT did not differ significantly between the seasons, although high intraseason variation of this process was noted. The laboratory experiment revealed that the size of lipid droplets was determined by photoperiod; SP induced 13-fold decrease, and continuous exposure to LP brought about a further 2.5-fold increase in the size of lipid droplets. Conversely, a significant decrease in lipid peroxidation was seen in LP bank voles in comparison with the SP animals. The data indicate that short photoperiod is responsible for the small size of lipid droplets in the BAT of bank voles during winter, which may be a necessary requirement for high thermogenic capacity of the tissue. Photoperiod appears also to affect lipid peroxidation in the BAT of these animals.

## Introduction

The mammals, living in temperate zone, usually show seasonal changes in their morphology, physiology, and behavior to cope with altering environmental conditions (Bartness et al. 2002; Concannon et al. 2001; Demas et al. 2002; Merritt et al. 2001). The most difficult season is winter, when ambient temperature decreases much below zero and food availability is restricted, especially for small mammals because of their adverse surface/volume ratio. To survive winter some species use daily torpor, and others enter hibernation (Heldmaier et al. 2004). Some species, such as bank vole, remaining active throughout the year, have to increase heat production for thermoregulation to maintain body temperature at normothermia.

In small mammals, an endogenous heat production is mainly achieved through nonshivering thermogenesis (NST), and brown adipose tissue (BAT) is the main site for NST (Cannon and Nedergaard 2004; Cinti 2005; Ricquier and Bouillaud 2000). This tissue shares the unusual property of accumulation and release of fatty acids, which are at the base of its activity (Cinti 2005). The lipid stores in brown adipocytes are accumulated as triglycerides. They are recruited after BAT activation via noradrenaline from sympathetic nervous system when basal metabolic heat production performed by internal organs is insufficient to maintain constant body temperature. Noradrenaline initiates triglycerides breakdown to free fatty acids that are both the substrate for thermogenesis and the regulators of UCP1 activity, the protein that allows heat production by uncoupling oxidative phosphorylation (Cannon and Nedergaard 2004; Golozubova et al. 2006).

It is well documented that most small mammals increase their NST capacity in winter or winter-like conditions (Li and Wang 2005a,b; Merritt and Zegers 1991; Wang et al. 2006a,b). This enhancement of efficiency of heat production is usually associated with increase in total amount of protein UCP1 and UCP1 messenger RNA level, cytochrome-c oxidase activity, and the total amount of mitochondrial protein (Demas et al. 2002; Klaus et al. 1988; Li and Wang 2005a; Praun et al. 2001; Wang et al. 1999, 2006b; Zang and Wang 2007). Furthermore, an increase in the total BAT weight accompanied by an increase in cellularity contribute to the enhanced oxidative capacity of this tissue (Cannon and Nedergaard 2004; Cinti 2005; Zang and Wang 2007).

Although seasonal regulation of BAT function is well demonstrated in several wild species under laboratory conditions, only few data on seasonal adjustment in small wild mammals are available. Klaus et al. (1988) observed, for example, that wild bank voles showed increased thermogenic properties of BAT during winter, but their brown fat mass remained seasonally constant. So far, it is unknown what the structure of brown fat is, especially lipid droplet size that appears to affect lipase activity and lipid bioavailability (Favé et al. 2004; McClements et al. 2009). Thus, it cannot be ruled out that seasonal changes in thermogenic capacity of BAT in the bank vole may also be associated with the changes in lipid droplet size in this tissue. Therefore, the main purpose of the present work was to examine seasonal changes in lipid droplet size in the BAT of wild bank voles. Because photoperiod is a critical environmental cue that triggers many seasonal responses in animals and even 60 % of the seasonal enhancement in NST may be induced by short day length (Heldmaier et al. 1989; Wang et al. 1999), the influence of photoperiod on the size of lipid droplets in the BAT of bank voles raised under laboratory conditions was also determined. In addition, lipid peroxidation was measured to find out whether oxidative stress occurs in the BAT of bank voles.

## Materials and methods

### Effect of season

Forty-four bank voles were captured in live traps in the Knyszyn Old Forest near Bialystok (northeastern Poland) from April 2006 to March 2007. They were transported to the laboratory on the same day and immediately euthanized. The animals were assigned into four seasonal groups according to the month of capture: (1) spring, March–May; (2) summer, June–August; (3) autumn, September–November, and (4) winter, December–February.

### Effect of photoperiod

Male bank voles, being F1 offspring of the wild-caught stock were used in the study. The animals were housed individually in stainless-steel cages (40 × 25 × 15 cm) lined with peat as an absorptive material and hay in the nest compartment at a constant temperature (19 ± 1°C) and 50–70 % relative humidity. They received ad libitum tap water and whole wheat grains. In addition, an identical amount of apple was offered to all animals (3 g/week) and was eaten completely. The food intake was measured throughout the experiment.

The bank voles were maintained under long photoperiod (16 h light/8 h dark) from the birth. After a 12-week period, six individuals were euthanized [the start-time group (LP-12)]. The remaining animals were randomly assigned into two groups according to photoperiod: (1) LP—the bank voles maintained under long photoperiod all the time and (2) SP—the animals transferred to short photoperiod (8 h light/16 h dark) for 6 weeks. At the end of 3- and 6-week photoperiod exposure, six individuals from each photoperiod group were euthanized.

### Assays

The animals were euthanized by cervical dislocation and weighed. Interscapular BAT was removed and weighed. A portion of BAT for histological examination was transferred to formalin. The remaining portion of the tissue was frozen and kept at −80°C until biochemical analysis.

### Histological examination and lipid droplet size measurement

One pad of the BAT from each animal was fixed in 4 % phosphate-buffered formalin, dehydrated in ethanol and xylene, embedded in paraffin, cut into 5-μm sections, and stained with hematoxylin and eosin for microscopic examination. The size of lipid droplets was measured as an area (μm^2^) (*n* = 50/vole) using MultiScanBase v. 14.02.

### Lipid peroxidation assay

Lipid peroxidation was assessed by measuring malondialdehyde formation, using the thiobarbituric acid (TBA) assay (Ohkawa et al. 1979). After thawing, a portion of the BAT was transferred to 1.0 ml chilled 0.25 M sucrose and homogenized with Teflon pestle in a glass homogenizer. A volume of 0.2 ml of 8.1 % sodium dodecyl sulfate, 1.5 ml of 20 % acetic acid, 1.5 ml of 0.8 % TBA, and 0.6 ml distilled water to 0.2 ml of the tissue homogenate were added and vortexed. The reaction mixture was placed in a water bath at 95°C for 1 h. After cooling, 1.0 ml of distilled water and 5.0 ml of butanol/pyridine mixture (15:1 *v*/*v*) were added and vortexed. After centrifugation, the absorbance of the organic phase was determined at 532 nm. Tetraethoxypropane was used to prepare a calibration curve. The results were expressed as TBA reactive substances (TBARS) (nanomoles/gram wet weight).

### Statistical analysis

Most of the data (body and BAT weights, lipid droplet size, and food intake) were expressed as mean ± SD. These values were analyzed by one-way analysis of variance (ANOVA) followed by the Duncan’s multiple-range test (SPSS 14.0). In the case of lipid peroxidation, the data were expressed as median and minimum–maximum values because of high intragroup variation. These data were analyzed by the non-parametric Kruskal–Wallis ANOVA with Mann–Whitney *U* test with the Bonferroni correction. Differences at *p* < 0.05 were considered statistically significant.

## Results

### Effect of season

The mean body weight of wild bank voles caught in spring and summer was significantly higher (35 %) than that of animals living in autumn and winter (*p* < 0.01) (Table 1), but no seasonal changes in the absolute weight of interscapular BAT were found in these animals. In consequence, the relative BAT weight (percent of body mass) in bank voles from autumn and winter (0.30 %) was significantly higher than that in the animals from spring and summer (0.18 %) (*p* = 0.01).

**Table 1:** Body and interscapular brown adipose tissue (BAT) weights, lipid droplet size, and lipid peroxidation (TBARS) in the wild bank voles caught in four seasons

Season	*n*	Body weight (g)	BAT weight (mg)	Lipid droplet size (μm^2^)	TBARS (nmol/g)
Spring	12	20.0 ± 3.0^a^	39 ± 18^a^	14.4 ± 6.1^ab^	805 (177–4,201)^a^
Summer	10	22.5 ± 2.1^a^	40 ± 21^a^	24.6 ± 4.9^a^	1,043 (256–2,225)^a^
Autumn	10	14.3 ± 1.9^b^	41 ± 13^a^	15.1 ± 14.7^ab^	294 (90–2,385)^a^
Winter	12	13.9 ± 2.3^b^	46 ± 16^a^	11.2 ± 7.6^b^	246 (176–426)^a^

Values represent means ± SD (body and BAT weights, lipid droplet size) or median and min–max values (TBARS). Values in the same column marked with different superscript letters are significantly different (*p* < 0.05)

**Fig. 1:**
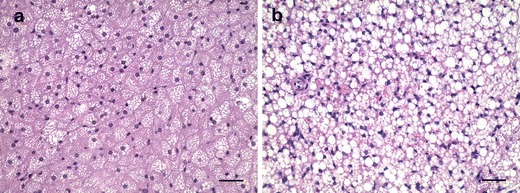
Representative photomicrographs of BAT section from the wild bank voles captured in **a** winter and **b** summer. Hematoxylin and Eosin staining. Scale bar = 30 μm

Histological examination revealed marked seasonal differences in lipid droplet size in the BAT of bank voles (Table 1). The smallest lipid droplets (mean, 11 μm^2^) were observed during winter (Fig. 1a), and an increase in size (by 30 %) was noted in spring. The biggest lipid droplets were found during summer (Fig. 1b)—over 2-fold higher in comparison to those in winter.

The nonparametric Kruskal–Wallis ANOVA revealed that lipid peroxidation (measured as TBARS) in the BAT of bank voles did not differ significantly among seasons (*H* = 5.76, *p* = 0.12; Table 1). However, high intraseason variation of this process was seen.

### Effect of photoperiod

Photoperiod affected significantly body weight of the bank vole (Table 2). The 3- and 6-week exposure to short photoperiod induced 15 and 30 %, respectively, decrease in the body weight in comparison to the respective LP animals. Furthermore, a significant decrease in food consumption in the SP-6 voles was detected (Table 2).

**Table 2:** Body and interscapular brown adipose tissue (BAT) weights, lipid droplet size, lipid peroxidation (TBARS), and food intake in the bank voles raised under long (LP) and short (SP) photoperiod

Age of animals (weeks)	Photoperiod exposure time (weeks)	Body weight (g)	BAT weight (mg)	Lipid droplet size (μm^2^)	TBARS (nmol/g)	Food intake (g/vole/day)
12	LP-12	24.5 ± 5.6^a^	177 ± 86^a^	107.1 ± 7.8^a^	242 (176–451)^a^	3.14 ± 0.25^a^
15	LP-15	25.0 ± 3.0^a^	245 ± 84^a^	128.3 ± 35.6^a^	41 (30–107)^b^	3.23 ± 0.59^a^
18	LP-18	26.6 ± 1.9^a^	220 ± 73^a^	266.1 ± 46.0^b^	51 (34 – 86)^b^	3.29 ± 0.51^a^
15	SP-3	21.2 ± 4.2^ab^	136 ± 128^a^	24.5 ± 20.2^c^	163 (118–656)^a^	3.28 ± 0.66^a^
18	SP-6	18.6 ± 2.0^b^	54 ± 17^b^	8.3 ± 3.0^c^	131 (40–216)^a^	2.70 ± 0.39^b^

Values represent means ± SD for *n* = 6 (body and BAT weights, lipid droplet size, food intake) or median and min-max values (TBARS). Values in the same column marked with different superscript letters are significantly different (*p* < 0.05). All bank voles were housed under long photoperiod from the birth. After 12 weeks a part of them was transferred to short photoperiod (SP), the remaining ones were housed under long photoperiod (LP) to the end of experiment

**Fig. 2:**
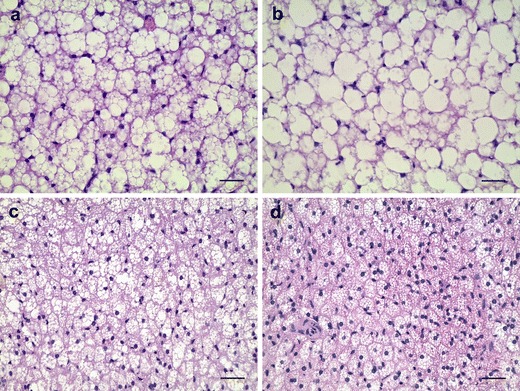
Representative photomicrographs of BAT section from the bank voles raised in laboratory under long (LP) and short (SP) photoperiod. **a** Bank voles kept under long photoperiod from the birth (start of the experiment) (LP-12). **b** Continuous exposure to LP for 6 weeks induced marked increase in lipid droplet size (LP-18). **c** Transferring the animals to short photoperiod for 3 weeks induced significant decrease in lipid droplet size but without changes in the tissue weight (SP-3). **d** After 6 weeks of SP exposure, further decrease in lipid droplet size was accompanied by a decrease in the absolute BAT weight (SP-6). Hematoxylin and eosin staining. Scale bar = 30 μm

The 3-week exposure of bank voles to short day length induced significant decrease in the lipid droplet size (77 %) (Fig. 2) but without changes in the absolute weight of BAT (Table 2). A further decrease in the size of lipid droplets (92 %) and concurrent 3-fold reduction of the BAT mass was recorded in the animals kept for 6 weeks under SP (Fig. 2, Table 2). In contrast, in the BAT of voles kept continuously under LP, an additional 2.5-fold increase in the size of lipid droplets was seen. Notably, lipid peroxidation in the BAT of the same animals was significantly lower than that in bank voles kept under short photoperiod (Table 2).

## Discussion

The results of the present study confirm previous observation (Klaus et al. 1988) that the absolute BAT weight in wild bank voles remains seasonally constant (Table 1). However, a lipid droplet size (fat content) in this tissue appears to undergo significant seasonal changes; the animals captured in winter display the smallest lipid droplets in their BAT, while the mean surface of droplets increases during spring, reaching the highest size in summer (Fig. 1, Table 1). The laboratory experiment performed in this study revealed further that short photoperiod is responsible for the smallest size of lipid droplets in the BAT of bank voles living in winter (Fig. 2, Table 2). This is evidenced by the fact that transferring the voles from a long to short photoperiod for 6 weeks caused a 90 % reduction in lipid droplet size, while continuous long photoperiod exposure induced a further enlargement of these droplets. Thus, a short photoperiod induces decrease in lipid droplet size in BAT, which may be a necessary requirement for high thermogenic capacity of the tissue in winter. Such a reduction in size of lipid droplets has been shown to enhance their accessibility to lipolytic enzymes and increase lipase activity (Favé et al. 2004; McClements et al. 2009), thereby raising thermogenic capacity of the tissue. Based on the results from the present work one can also conclude that there are two phases of short photoperiod action on the BAT. In the first phase (after 3 weeks of short photoperiod exposure), a 4-fold decrease in lipid droplet size is observed but without changes in the total BAT weight (Fig. 2c, Table 2). In the second one (after 6 weeks of short day acclimation), a further decrease in lipid droplet size is accompanied by the reduction of BAT mass. Thus, it is reasonable to assume that in the first phase only a breakage of lipid droplets occurs, and in the second one, the intracellular triglycerides stores are utilized for thermogenesis. However, further investigations are needed to clarify the mechanism by which short photoperiod induces the decrease in lipid droplet size.

Although photoperiod appears to be a key factor determining lipid droplet size in the BAT of bank voles, also other factors such as ambient temperature, quality and quantity of food as well as general life conditions may affect fat content in the tissue of wild animals. Thus, a 10-fold smaller lipid droplets in the BAT of wild bank voles from summer as compared to those raised in laboratory under long photoperiod (Tables 1 and 2) could result, for instance, from a higher energy expenditure associated with food searching and other physical activities in the field than in the cage.

It has been shown that the exposure of small mammals to cold induces nonshivering thermogenesis in the BAT (Wang et al. 1999, 2006a,b; Zang and Wang 2007). During this process, there is several fold increase in oxygen consumption, which is necessary for the oxidation of fatty acids in the mitochondria (Zaninovich et al. 2002). One consequence of this high aerobic metabolism is heat production; another one is a concurrent increase in the generation of reactive oxygen species (ROS), which can oxidize various cellular macromolecules, including lipids (Barja de Quiroga 1992). It is known from the studies on rats that lipid peroxidation in the BAT is effectively prevented by antioxidant system (Włostowski et al. 2008). In contrast, lipid peroxidation in the wild bank voles and those raised in laboratory under short photoperiod was relatively high and variable (Tables 1 and 2). These data suggest that an antioxidant system in the BAT of bank voles is unable to efficiently prevent lipids, specifically unsaturated fatty acids (UFA) from peroxidation. However, no histopatological changes occurring in the tissue, even at lipid peroxidation as high as 4,000 nmol TBARS/g (Table 1), indicate that UFA building bilayer of the cellular membranes are probably not the target for ROS attack. Instead, a good target for this attack may be UFA originating from lipid droplets during the activation of BAT. Thus, it cannot be excluded that a fraction of UFA in the cytoplasma may play a role as free radical scavengers, thereby protecting other vital macromolecules against oxidative damage. Still, this hypothesis requires verification in further studies.

The present work also demonstrates that the mass of adult bank voles, transferred to short photoperiod for 6 weeks, undergoes significant regression (30 %) and concurrently the food intake decreases by about 20 % (Table 2). Because no effect of cold on the body weight of bank voles has been detected (Włostowski et al. 1996), therefore, short photoperiod appears to be a major factor responsible for lowering body mass in the wild bank voles during winter (Table 1) (Klaus et al. 1988; Włostowski et al. 1988). These results confirm the notion that winter decline in body weight is an important mechanism for the reduction of energy requirement when food availability is restricted and cold stress occurs (Lovegrove 2005).

In conclusion, photoperiod appears to determine lipid droplet size in the BAT of bank voles; specifically, short photoperiod induces decrease in their size, which may be a necessary requirement for high thermogenic capacity of the tissue in winter. Photoperiod also affects lipid peroxidation in the BAT, but the high intraseason variation of this process remains to be clarified.

### Ethics

All experimental procedures were approved by the Local Ethical Committee (Medical University of Bialystok) and were compatible with standards of the Polish Law on Experimenting on Animals, which implements the European Communities Council Directive (86/609/EEC).
